# Galectin-3 levels relate in children to total body fat, abdominal fat, body fat distribution, and cardiac size

**DOI:** 10.1007/s00431-017-3079-5

**Published:** 2018-01-11

**Authors:** Magnus Dencker, Daniel Arvidsson, Magnus K. Karlsson, Per Wollmer, Lars B. Andersen, Ola Thorsson

**Affiliations:** 1 Department of Medical Imaging and Physiology, Skåne University Hospital, Lund University, 205 02 Malmö, Sweden; 2 Center for Health and Performance, Department of Food and Nutrition, and Sports Science, University of Gothenburg, Gothenburg, Sweden; 3 Department of Clinical Sciences and Orthopaedics, Clinical and Molecular Osteoporosis Research Unit, Skåne University Hospital, Lund University, Lund, Sweden; 4 Department of Teacher Education and Sport, Sogn and Fjordane University College, Sogndal, Norway; 5 Department of Sports Medicine, Norwegian School of Sport Sciences, Oslo, Norway

**Keywords:** DXA, Body fat, Children, Galectin-3

## Abstract

Galectin-3 has recently been proposed as a novel biomarker for cardiovascular disease in adults. The purpose of this investigation was to assess relationships between galectin-3 levels and total body fat, abdominal fat, body fat distribution, aerobic fitness, blood pressure, left ventricular mass, left atrial size, and increase in body fat over a 2-year period in a population-based sample of children. Our study included 170 children aged 8–11 years. Total fat mass and abdominal fat were measured by dual-energy x-ray absorptiometry (DXA). Body fat distribution was expressed as abdominal fat/total fat mass. Maximal oxygen uptake was assessed by indirect calorimetry during a maximal exercise test and scaled to body mass. Systolic and diastolic blood pressure and pulse pressure were measured. Left atrial size, left ventricular mass, and relative wall thickness were measured by echocardiography. Frozen serum samples were analyzed for galectin-3 by the Proximity Extension Assay technique. A follow-up DXA scan was performed in 152 children 2 years after the baseline exam. Partial correlations, with adjustment for sex and age, between galectin-3 versus body fat measurements indicated weak to moderate relationships. Moreover, left atrial size, left ventricular mass, and relative wall thickness and pulse pressure were also correlated with galectin-3. Neither systolic blood pressure nor maximal oxygen uptake was correlated with galectin-3. There was also a correlation between galectin-3 and increase in total body fat over 2 years, while no such correlations were found for the other fat measurements.

*Conclusion*: More body fat and abdominal fat, more abdominal body fat distribution, more left ventricular mass, and increased left atrial size were all associated with higher levels of galectin-3. Increase in total body fat over 2 years was also associated with higher levels of galectin-3.

**Table:** 

**What is Known:**
• *Galectin-3 has been linked to obesity and been proposed to be a novel biomarker for cardiovascular disease in adults.*
• *Information on this subject in children is very scarce.*
**What is New:**
• *The present study demonstrates a relationship between galectin-3 levels and total body fat, abdominal fat, body fat distribution, cardiac size and geometry, and increase in total body fat over 2 years in young children.*

## Introduction

Galectin-3 is a protein that has been suggested to stimulate collagen production and hypertrophy as responses to injury through different mechanisms [1]. It has also emerged as a novel molecule for regulatory functions within the immune system [2, 3]. The potential role of galectin-3 as a circulating biomarker for cardiovascular diseases has been mainly tested in patients with heart failure [1, 4–6]. Galectin-3 levels have been shown to correlate with clinical outcomes in patients with atherothrombosis [7], and to correlate with traditional risk factors for cardiovascular diseases and with mortality in subjects from the general population [8]. Elevated galectin-3 levels have also been associated with increased risk for stroke after carotid endarterectomy [9]. In adults, galectin-3 has been shown to be elevated in obesity [10, 11]. As obese children and adolescents have an increased risk of developing adult obesity and are more likely to experience significant short- and long-term health problems [12–14], it is relevant to explore potential relationships between galectin-3 versus body fat and other markers for health at a young age. There are scarce data on this subject in children [15]. The purpose of this investigation was therefore to assess relationships between galectin-3 levels and total body fat, abdominal fat, body fat distribution, aerobic fitness, blood pressure, left ventricular mass, left atrial size, and also increase in body fat over 2 years in a community sample of children.

## Material and methods

### Subjects

Children were recruited from four different schools in Malmö, Sweden. The schools were all situated in socially homogeneous middle-class areas with inhabitants of essentially non-immigrant origin. All 477 children (boys = 259, girls = 218) attending third or fourth grade were invited to participate. Out of these, 248 accepted the invitation (boys = 140, girls = 108), and parents of 172 children gave consent to give blood sample. A complete data set with blood samples and other measures were available in 170 children (92 boys and 78 girls) giving an inclusion frequency of 36%. A 2-year follow-up DXA scan was available in 152 children (84 boys and 68 girls). A separate study of anthropometric data, from school nurses, for all children that received an invitation to participate in the study showed no significant differences in height, body mass, or BMI between the children that chose to participate and those who did not [16].

### Anthropometric measures

Anthropometric measures were performed at baseline and at the follow-up measurement (2.0 ± 0.1) years later. Height was measured to the nearest 1 cm using a fixed stadiometer (Hultafors AB, Hultafors, Sweden) and body mass was measured to the nearest 1 kg (Avery Berkel model HL 120, Avery Weigh-Tronix Inc., Fairmont, MN, USA). Puberty status was assessed by self-evaluation according to Tanner [17].

### Dual-energy x-ray absorptiometry

Body composition was measured by DXA total body scan (DPX-L version 1.3z, Lunar, Madison, WI, USA), both at baseline and at the follow-up measurement. Total body fat mass and abdominal fat were quantified. Percent body fat was calculated as percentage of total body mass. Body fat distribution was calculated as abdominal fat/total body fat mass. Studies have shown that DXA provides accurate measurements of fat mass, including regional fat mass [18].

### Measurement of aerobic fitness

Maximal oxygen uptake was determined by a maximum exercise test at the baseline. The test was performed on a bicycle ergometer (Rodby rhc, model RE 990, Rodby Innovation AB, Karlskoga, Sweden). Expired gas was analyzed for the concentration of O_2_ and CO_2_ (Sensor Medics 2900, SensorMedics Inc., Yorba Linda, CA, USA). All children used the same protocol with an initial workload of 30 W and an increase of 15 W per minute. Maximum heart rate and maximum respiratory exchange ratio were recorded. Maximal oxygen uptake was determined as the highest value during the last minute of exercise and scaled to body mass (ml/min/kg). The exercise test was considered acceptable if it met one of the following criteria: maximum respiratory exchange ratio ≥ 1.0, maximum heart rate > 90% of predicted value, or signs of intense effort [19].

### Blood pressure

A Dinamap pediatric vital sign monitor (model XL, Critikron Inc., Tampa, FL, USA) was used to measure systolic and diastolic blood pressure in the seated position after 15 min rest [20]. The mean of three measurements was used. Pulse pressure was calculated as the difference between systolic and diastolic blood pressure.

### Echocardiography

Echocardiographic examination was performed using Sonos 2500 (Philips Inc., Eindhoven, the Netherlands) or Aspen (Acuson Inc., Mountain View, CA, USA) and in accordance with the American Society of Echocardiography recommendations [21]. The following variables were measured: end-diastolic left ventricular diameter (LVDD), left atrial end-systolic diameter, end-diastolic inter-ventricular septum thickness (IVS), and end-diastolic posterior wall thickness (PW). Left ventricular mass was calculated using the American Society of Echocardiography convention: left ventricular mass = 0.83 × [(LVDD + IVS + PW)^3^ − LVDD^3^] + 0.6 (measurements in cm) [21]. Both left ventricular mass and left atrial size were indexed for height [22–24]. Relative wall thickness of the left ventricle was calculated as 2 × PW/LVDD [21].

### Blood samples

Non-fasting venous serum samples were drawn from 172 children and stored at − 70 °C until analysis. The samples were collected in conjunction with the DXA measurement. Galectin-3 was analyzed by the Proximity Extension Assay technique using the Proseek Multiplex CVD 96 × 96 reagents kit (Olink Bioscience, Uppsala, Sweden) at the Clinical Biomarkers Facility, Science for Life Laboratory, Uppsala, as previously described [25, 26]. The intra- and inter-assay variations were 9 and 12%, respectively. Data are presented as arbitrary units (AU). Values can be transformed to actual concentrations on the Olink Bioscience website. Two samples were excluded due to technical problems at the analysis.

### Statistical analyses

All analyses were made in Statistica 12 (StatSoft Inc., Tulsa, OK, USA). The descriptive data are presented as means ± SD. Differences in anthropometric data between boys and girls were tested using Student’s *t* test. Distributions of body fat measurements and abdominal fat were skewed and therefore normalized by natural logarithm. Regression analysis was used to calculate partial correlations for all children, with adjustment for sex and age, between the galectin-3 and total body fat, abdominal fat, percent body fat, body fat distribution, maximal oxygen uptake, systolic and diastolic blood pressure, pulse pressure, left ventricular mass, and left atrial size. Partial correlations were also calculated between biomarkers and change in total body fat, abdominal fat, percent body fat, and body fat distribution over the 2-year follow-up period, adjusted for sex, age, and change in Tanner stage. A value of *P* < 0.05 was regarded as statistically significant.

## Results

Children that did not consent to give a blood sample were slightly younger than those who gave consent (9.6 ± 0.6 vs. 9.9 ± 0.6 years, *P* = 0.002). No differences were found in anthropometric, fitness, or DXA data between those children who were part of the final study group at baseline and those who were not (data not shown). The children who did not participate in the follow-up were slightly younger at baseline than those who did (9.7 ± 0.6 vs. 9.9 ± 0.6 years, *P* = 0.005). There were no differences in anthropometrics, fitness, or DXA data between those who participated in the follow-up and those who did not (data not shown). At baseline, three girls were Tanner stage 2; all other children were Tanner stage 1. The distributions of Tanner score at follow-up were (N for stage 1 = 18, stage 2 = 63, stage 3 = 49, stage 4 = 22). Galectin-3 was borderline significantly related to change in Tanner stage (*r* = − 0.15, *P* = 0.064), and partial correlations for the follow-up measurements were adjusted for change in Tanner stage. At baseline, girls had more total body fat and abdominal fat, higher percent body fat, lower maximal oxygen uptake, and less left ventricular mass than boys. At follow-up, boys had a higher increase in percent body fat. Girls had higher Tanner score than boys at follow-up. Summary of baseline and follow-up data is displayed in Table 1. Galectin-3 was associated with age (*r* = − 0.17, *P* < 0.05) and partial correlations were therefore adjusted for age. Regression analysis indicated weak to moderate relations between galectin-3 and total body fat, abdominal fat, percent body fat, body fat distribution, left ventricular mass, left atrial size, relative wall thickness, diastolic blood pressure, and pulse pressure. No such correlation was found for systolic blood pressure and maximal oxygen uptake (Table 2). Weak correlation existed between galectin-3 and increase in total body fat over 2 years but not with increase in abdominal fat, percent body fat, or change in fat distribution (Table 2).

**Table 1:** Display of age, anthropometric, fitness, and Tanner statistics (± SD) for baseline and follow-up measurements. Galectin-3 in arbitrary units (AU)

Variable	Boys (*n* = 92)	Girls (*n* = 78)	*P* value
Baseline			
Age (year)	10.0 ± 0.6	9.8 ± 0.6	0.03
Height (cm)	141 ± 7	141 ± 7	0.69
Body mass (kg)	35.2 ± 7.4	34.6 ± 6.6	0.57
BMI (kg/m^2^)	17.5 ± 2.6	17.4 ± 2.7	0.90
Total body fat (kg)	6.2 ± 4.6	8.0 ± 4.6	0.01
Percent body fat (%)	16.1 ± 8.3	22.0 ± 8.5	< 0.001
Abdominal fat (kg)	2.3 ± 2.0	3.2 ± 2.2	0.01
Fat distribution (abdominal fat/total body fat)	0.36 ± 0.04	0.37 ± 0.05	0.09
Fitness (ml/min/kg)	42 ± 7	36 ± 6	< 0.001
Maximum heart rate (beats/min)	189 ± 14	184 ± 16	0.03
Respiratory exchange ratio	1.0 ± 0.1	1.0 ± 0.1	0.64
Systolic blood pressure (mmHg)	104 ± 8	104 ± 9	0.75
Diastolic blood pressure (mmHg)	60 ± 5	60 ± 6	0.48
Pulse pressure (mmHg)	44 ± 8	43 ± 7	0.29
Left ventricular mass (g/m)	53.9 ± 12.0	48.3 ± 11.3	0.002
Left atrial diameter (mm/m)	19.9 ± 2.4	19.5 ± 2.0	0.22
Galectin-3 (AU)	4.34 ± 0.31	4.44 ± 0.55	0.12
Tanner stage score	1.0 ± 0.0	1.0 ± 0.2	0.06
Follow-up	Boys (*n* = 84)	Girls (*n* = 68)	
Age (year)	11.9 ± 0.6	11.8 ± 0.6	0.16
Height (cm)	152 ± 8	153 ± 8	0.31
Body mass (kg)	43.2 ± 8.6	43.9 ± 9.1	0.62
BMI (kg/m^2^)	18.5 ± 2.8	18.6 ± 3.2	0.97
Increase in total body fat (kg)	2.1 ± 2.6	2.2 ± 2.4	0.95
Increase in abdominal fat (kg)	1.0 ± 1.2	1.1 ± 1.5	0.48
Increase in percent body fat (%)	1.4 ± 4.0	0.0 ± 3.8	0.03
Change in fat distribution	0.02 ± 0.03	0.03 ± 0.15	0.32
Tanner stage score	2.3 ± 0.9	2.7 ± 0.8	0.007

**Table 2:** Partial correlations and *P* values, adjusted for sex and age for baseline measurements, between galectin-3 versus different baseline measurements and change in body fat measurements at follow-up, adjusted for sex, age, and change in Tanner score

Variable	*n* = 170
Baseline	
Height	0.11 (0.12)
Body mass	*0.28 (< 0.001)
BMI	*0.29 (< 0.001)
Total body fat	*0.31 (< 0.001)
Percent body fat	*0.30 (< 0.001)
Abdominal fat	*0.30 (< 0.001)
Body fat distribution	*0.20 (0.01)
Fitness	− 0.11 (0.15)
Systolic blood pressure	0.03 (0.69)
Diastolic blood pressure	*− 0.16 (0.03)
Pulse pressure	*0.16 (0.04)
Left ventricular mass	*0.31 (< 0.001)
Left atrial diameter	*0.17 (0.03)
Relative wall thickness	*0.18 (0.02)
Follow-up	*n* = 152
Increase in TBF	*0.16 (0.04)
Increase in AFM	0.14 (0.09)
Increase in percent body fat	0.01 (0.88)
Change in body fat distribution	− 0.01 (0.87)

*Highlights significant (*P* < 0.05) correlations

## Discussion

The present study is the first to demonstrate weak to moderately strong associations between galectin-3 levels and total body fat, abdominal fat, body fat distribution, cardiac size and geometry, and increase in total body fat in a population-based sample of young children. These findings may suggest a role of galectin-3 in cardiovascular risk factor stratification in children, which has not been established previously.

The role of galectin-3 has mainly been investigated in adult patients with heart failure [1, 4–6]. In children, galectin-3 has previously been investigated as an inflammatory marker in chronic hepatitis B infection [27], juvenile idiopathic arthritis [28], or brain neoplasms [29]. We are aware of only one investigation in unselected children that has explored potential relations with galectin-3 and risk factors for diseases [15]. Meeusen and co-workers studied 240 children aged 2–17 years. No sex differences in galectin-3 levels were found, which agrees with our finding. However, this study found no relationship between galectin-3 levels and BMI, which is in disagreement with our findings of consistent relationships between galectin-3 levels and all the body fat measurements. One could only speculate of reasons for these discrepancies, but with participants from age 2–17 years, a significant amount of noise may be introduced with puberty. It is also known that BMI cannot differentiate between muscle and fat mass. There has been significant debate of the connection between obesity and systemic inflammation [30–33]. In adults, galectin-3 has been shown to be elevated in obesity [10, 11], and we confirm a relation to body fat measurements in an unselected population of children. The exact mechanisms behind our findings are not known. Galectin-3 has a variety of regulatory functions within the immune system [2, 3] and is known to be produced by adipose tissue and involved in adipose tissue growth and maturation [34, 35]. Moreover, galectin-3 levels are higher in subjects with type 2 diabetes and have been suggested to have a role from prediabetes to type 2 diabetes [11, 36]. The proposed pathways include galectin-3 participation in the adipocytes ability to respond with enlargement to fat overload after a high-fat diet [36].

Based on studies in heart failure patients [1, 4–6], galectin-3 was recently recommended as an additive for risk stratification in heart failure patients [37]. Galectin-3 has been shown to stimulate cardiac collagen production and hypertrophy as through a number of different mechanisms [1]. Our finding of relationships between galectin-3 and left ventricular mass, left atrial size, and relative wall thickness is interesting and novel. There was only a modest relationship between pulse pressure and galectin-3, which could suggest that the observed correlations between galectin-3 and cardiac size and geometry are in part mediated by blood pressure. The finding that diastolic blood pressure was associated in an opposite direction than hypothesized could be a random finding. There was no significant relation between galectin-3 and fitness, although the relation was in the hypothesized direction. This could be a stochastic finding and a larger study with more statistical power may give a more definitive answer.

Major strengths of the present investigation are the urban community-based sample, the multitude of measurements, and objective measurement of body fat by DXA. The inclusion frequency in this study of 52% might be considered somewhat low. A complete data set was available in no more than 36% (170 out of 477 invited children). However, a separate study of anthropometric data from all children that received an invitation to participate in the study showed no significant differences in height, body mass, or BMI between the children that chose to participate and those who did not [16]. Moreover, there were no major differences (except a slight age difference) in baseline measurements between those who participated in the follow-up versus those who did not. This suggests that the final study group was fairly representative. The fact that we had non-fasting blood samples has not biased the analysis as it has been shown that galectin-3 levels are not altered by food intake [38].

## Conclusions

The present study establishes a weak to moderate relationship between galectin-3 levels and total body fat, abdominal fat, body fat distribution, cardiac size and geometry, and increase in total body fat over 2 years in unselected young children.

## Abbreviations

AU: Arbitrary units
DXA: Dual-energy x-ray absorptiometry
IVS: End-diastolic inter-ventricular septum thickness
LVDD: End-diastolic left ventricular diameter
PW: End-diastolic posterior wall thickness
